# Exempting low-risk health and medical research from ethics reviews: comparing Australia, the United Kingdom, the United States and the Netherlands

**DOI:** 10.1186/s12961-019-0520-4

**Published:** 2020-01-28

**Authors:** Anna Mae Scott, Simon Kolstoe, M. C. ( Corrette) Ploem, Zoë Hammatt, Paul Glasziou

**Affiliations:** 1 Institute for Evidence-Based Healthcare, Bond University, 14 University Drive, Robina, QLD 4226 Australia; 2 University of Portsmouth, Portsmouth, UK; 3 Amsterdam University Medical Centre, Amsterdam, The Netherlands; 4 Z Consulting LLC, Denver, USA; 5 John A. Burns School of Medicine, University of Hawaii, Honolulu, USA

**Keywords:** Research ethics, research committees, waste in research, low-risk research, exemption, international variation, human, health research, medical research

## Abstract

**Background:**

Disproportionate regulation of health and medical research contributes to research waste. Better understanding of exemptions of research from ethics review in different jurisdictions may help to guide modification of review processes and reduce research waste. Our aim was to identify examples of low-risk human health and medical research exempt from ethics reviews in Australia, the United Kingdom, the United States and the Netherlands.

**Methods:**

We examined documents providing national guidance on research ethics in each country, including those authored by the National Health and Medical Research Council (Australia), National Health Service (United Kingdom), the Office for Human Research Protections (United States) and the Central Committee on Research Involving Humans (the Netherlands). Examples and types of research projects exempt from ethics reviews were identified, and similar examples and types were grouped together.

**Results:**

Nine categories of research were exempt from ethics reviews across the four countries; these were existing data or specimen, questionnaire or survey, interview, post-marketing study, evaluation of public benefit or service programme, randomised controlled trials, research with staff in their professional role, audit and service evaluation, and other exemptions. Existing non-identifiable data and specimens were exempt in all countries. Four categories – evaluation of public benefit or service programme, randomised controlled trials, research with staff in their professional role, and audit and service evaluation – were exempted by one country each. The remaining categories were exempted by two or three countries.

**Conclusions:**

Examples and types of research exempt from research ethics reviews varied considerably. Given the considerable costs and burdens on researchers and ethics committees, it would be worthwhile to develop and provide clearer guidance on exemptions, illustrated with examples, with transparent underpinning rationales.

## Introduction

In 2009, it was estimated that 85% of research funding for biomedical science (including clinical, health services and basic science) research is avoidably wasted, at an annual cost of approximately $170 billion [1–3]. This waste is due to the failure to ask questions relevant to users of research, appropriately design, conduct and analyse research, efficiently regulate and manage research, produce accessible and full research reports, and produce unbiased and useable research reports [2]. The inefficient regulation and management of research has been argued to manifest itself in the hyper-regulation of research, that is, regulation, governance and management, that are burdensome and disproportionate to the risks posed by the research to the participants [4].

Nearly all health and medical research involving humans is generally required to undergo a review for adherence to ethical standards and guidelines in terms of the balance of benefits and harms, rights and well-being of participants (hereafter ‘ethics review’). Where research participation involves a considerable physical risk, psychological risk or burden to participants, ethics reviews are an important means of protecting the health and well-being of research participants. However, some categories of ethics reviews have been criticised for impeding or delaying research [5–7], imposing costs that do not contribute to greater protection of research participants [8], being overly burdensome [9], and inconsistent risk determinations for the same study protocol across multiple sites [10–13].

The last of these seems to have been especially problematic in relatively simple studies, where the likelihood of discomfort, harm or inconvenience to participants is not expected to exceed that encountered in daily life, or whilst undergoing routine examinations or tests [11]. For example, a low-risk survey of medical students deemed exempt from further review by its home (United States-based) institution nevertheless required further reviews by 84 other Institutional Review Boards in United States medical schools [14]; a minimal risk health services research project required a full board review by 15 sites and was granted expedited review by two [15]. This variability can impose considerable costs – over 50 person-months costing $120,000 USD in the first case, and 27 months costing $170,000 USD in the second. The issue is not specific to the United States, as an Australian multi-site study on the quality of hospital care reported spending over $263,000 AUD in 1 year on costs associated with the varying institutional consent processes [16].

‘Proportional ethics review’ may be one way to help mitigate this problem. Proportional review pins the level of review and scrutiny to the level of perceived risk associated with the proposed research project – research projects that carry no or low risk receive no or minimal review; projects which carry higher risk, conversely, undergo more extensive review. The attractiveness of this approach consists in its efficiency – “*each application would receive no more scrutiny than it needs*” [17]. The implication of this approach is that certain types of low or negligible risk human health/medical research could be exempted entirely from ethics reviews. ‘Exemption from ethics review’ can include a range of approaches. For example, it could involve bypassing review by an ethics committee completely before commencing the research project, informing the committee of the project (e.g. by submitting an exemption form) but not submitting an application for review, or some form of partial or expedited review (e.g. by a sub-committee of the ethics committee or solely by its chair).

Comparisons between different countries’ approaches to exempting from ethics reviews of low-risk human health/medical research – that is, research that generally falls on the minimal burden, intrusion or inconvenience end of the research risk spectrum – may shed light into each country’s individual practices as well as identify possible solutions to problems [18–20]. Better understanding of the examples of exemptions of human health/medical research from ethics review in different jurisdictions may also help guide modification of review processes and help reduce waste of researchers’ and ethics committees’ time and resources when assessing low risk research.

To increase understanding of these issues we have attempted to identify explicit examples of exemptions of low-risk human health/medical research from ethics reviews in Australia, the United Kingdom, the United States and the Netherlands.

## Methods

For the purposes of this project, we considered ‘exempt from ethics review’ to be those types or examples of research that were explicitly identified as such. For example, in the United Kingdom, this included research projects that were exempted from a requirement to undergo review by a National Health Service (NHS) research ethics committee; in the United States, ‘exempt’ projects were those that fell outside the Code of Federal Regulations Title 45: Public Welfare, part 46 (45 CFR 46). (In practice, these two approaches would be considered ‘bypassing ethics review altogether’ and ‘informing the committee but not applying for ethics review’ end of the spectrum).

Australia, the United Kingdom, the United States and the Netherlands were selected as comparator countries because of the anticipated breadth of examples, availability of experts with knowledge of the key features of each jurisdiction’s health and medical research milieu as well as its research ethics systems (SK, CP, ZH), and the availability of documentation in English.

The analysis was document based. To identify examples or research project types that were ‘exempt from ethics review’, we examined documents providing national guidance on research ethics in each country. We focused on national level guidance to ensure comparability. The analysed documents were the following:

1. Australia: National Health and Medical Research Council National Statement on Ethical Conduct in Human Research 2007 (Updated 2018) [21]
2. United Kingdom: NHS Governance Arrangements for Research Ethics Committees: 2018 Edition” [22] and NHS Standard Operating Procedures for Research Ethics Committees version 7.3 (September 2018) [23]
3. United States: 45 CFR 46 section 104 [24] and Department of Health and Human Services, Office for Human Research Protections, Human Subject Regulations Decision Charts [25]
4. Netherlands: Central Committee on Research Involving Humans (CCMO), Your Research: Is it Subject to WMO [Medical Research Involving Human Subjects Act] or Not? [26]

One author (AMS) read all documents in full and identified types and examples of human health/medical research explicitly identified in those documents as being exempt from ethics review. A table was created with a column for each country, and all identified exemptions were listed in the table, creating as many rows as required (one for each exemption). These were then reorganised, and categories of exemptions that were similar or shared across countries were placed in the same row. This was done iteratively, until no more similar categories of exemptions were identified. Where uncertainties arose regarding interpretation or terminological differences regarding any of the exemptions, the expert on that jurisdiction (SK, CP, ZH) was queried. Once uncertainties were resolved, the final table with exemptions was generated by two authors (AMS, PG).

## Results

The overall results (Table 1) illustrate considerable variation in countries’ approaches to exempting research from ethics review. Across all four countries, almost half of the categories had some examples of exemptions. Existing non-identifiable data and specimens were exempt in all countries. Four categories – evaluation of public benefit or service programme, randomised controlled trials, research with staff in their professional role, and audit and service evaluation – were exempted by one country each. The Netherlands had the most extensive exemptions.

**Table 1:** Examples and types of health and medical research exempted from ethics reviews

Exemption	Australia	United Kingdom	United States of America	Netherlands
Existing data/specimen	Existing collection of data or records that contain only non-identifiable data	Previously collected, non-identifiable tissue samples	Secondary research use of identifiable biospecimens (subject to criteria)	Exempt
Questionnaire or survey	Filling in a form, participating in a street survey (where those are no more than inconvenience)		Educational tests, survey procedures (subject to criteria)	Exempt, unless it imposes rules of behaviour (e.g. required to be done repeatedly) or topic is sensitive (e.g. suicide, abortion)
Interviews			Interview procedures (subject to criteria)	Exempt, unless it imposes rules of behaviour (e.g. required to be done repeatedly) or topic is sensitive (e.g. suicide, abortion)
Post-marketing studies		Healthcare market research conducted by professional market researchers		e.g. collecting data on adverse effects of medicines already available on the market
Evaluation of public benefit or service programme			e.g. procedures for obtaining benefits, alternatives, changes in payment levels	
Randomised controlled trials				May be exempt if meets the exemption rule below (see ‘other’ category)
Research with staff in their professional role		Research involving NHS staff acting in their professional roles		
Audit and service evaluation	^a^	Audit and service evaluation		
Other exemptions	“*Giving up time to participate in research*” (example of an inconvenience)			Medical/scientific research that does not expose persons to treatment, or require persons to follow certain behavioural rules

^a^ Dealt with outside of the National Statement (see below)

### Australia

The National Health and Medical Research Council’s National Statement on Ethical Conduct in Human Research 2007 (Updated 2018) [21] identifies human health and medical research that can be exempted from ethics review in Australia.

Section 5.1.22 allows institutions to exempt from ethics review research that is:

1. Negligible risk research and
2. Involves the use of existing collections of data or records that contain only non-identifiable data about human beings

Negligible risk is defined in s2.1.7 as research where “*any foreseeable risk is no more than inconvenience*”. ‘Inconvenience’ is the lowest of the three types of risks identified by the National Statement; ‘discomfort’ is classified as a higher level of risk, and ‘harm’ is classified higher still. Inconvenience “*may include filling in a form, participating in a street survey, or giving up time to participate in research*” [21].

According to s 5.1.22, therefore, exempt research in Australia is research that involves at most ‘inconvenience’ (i.e. research that involves completing a form, participating in a street survey, giving up time to take part in research), and research involving existing data/records on human beings who are non-identifiable.

It is worth emphasising that the National Health and Medical Research Council (NHMRC) National Statement focuses on ‘research.’ Because quality assurance and evaluation activities may not be considered research, they are addressed by a separate NHMRC document [27]. Because they commonly involve “*minimal risk, burden or inconvenience to their participants*”, the ethics review processes are not thought to be optimal, but rather that “*organisational oversight*” ought to apply [27].

### United Kingdom

The United Kingdom has the largest coordinated NHS in the world. The NHS constitution commits the NHS to “*innovation and to the promotion, conduct and use of research*” [28]. This provides clear justification for the use of audit, service development and research to advance both medical knowledge and standards of care.

Audit and service evaluation are sometimes differentiated from research; whereas ‘research’ encompasses activities aiming at creating new, generalisable knowledge, ‘audits’ review the current care practice or care delivery and ‘quality improvement’ (sometimes also called service evaluation) pilots new practices [11, 29]. Because both audit and service evaluation are considered part of usual professional practice in the NHS, they are exempt from oversight processes that govern research – that is, they do not require review by a research ethics committee (REC) as any ethical concerns should be identified as inherent in clinical or professional ethics [30].

Research involving NHS staff is also exempt within the NHS system. This decision was based on legal advice that any ethics issues that arise (e.g. surrounding consent, risks, etc.) are already covered by employment law [22].

Research limited to work on material such as previously collected cells or tissues that are non-identifiable is generally exempted from REC review [22].

Healthcare market research does not normally require a REC review, as long as it is conducted by professional market researchers and complies with the guidelines of the British Healthcare Business Intelligence Association [30].

Nevertheless, as a matter of law, any work in the United Kingdom – whether audit, service evaluation or staff research – that falls under definitions contained within the Medicines for Human Use (Clinical Trials) Regulations 2004, Medical Devices Regulations 2002, Mental Capacity Act 2005, and Human Tissue Act 2004, must be reviewed by a United Kingdom Ethics Committee Authority-authorised REC [22]. In addition to studies requiring REC review by law, all other projects that have been defined as research and involve NHS patients or facilities must be reviewed by a REC. For non-NHS work (e.g. at universities, companies or with private patients), the aforementioned acts and regulations remain applicable, and any relevant work must be reviewed by a United Kingdom Ethics Committee Authority-recognised committee although, on occasion, healthcare research not subject to these acts may be conducted with ethics reviews provided by university or other RECs.

### United States

Research conducted or supported by the United States Department of Health and Human Services is regulated by the United States Code of Federal Regulations. ‘Exempt research’ is research for which there is no submission for ethics review; instead, an “*exempt study submission form*” is submitted to an Institutional Review Board (IRB) [24, 25].

Research eligible for exemption under United States regulation 45 CFR 46 (revised July 2018) includes secondary research uses of identifiable biospecimens as long as at least one of four criteria are met, namely (1) the research plan is publicly available; (2) the identity of human ‘subjects’ cannot be readily ascertained; (3) it involves information collection and analysis for ‘healthcare operations’, ‘research’ or ‘public health activities and purposes’; or (4) the research is being performed by or on behalf of a federal department or agency using information generated or collected by the government for non-research activities.

Research involving educational tests (e.g. cognitive, diagnostic, aptitude), survey procedures, and interview procedures is also exempt if at least one of three criteria are met, namely (1) the identity of human ‘subjects’ cannot be readily ascertained; (2) disclosure of responses outside the research would not place ‘subjects’ at risk of criminal or civil liability, or be damaging to financial standing, employability, educational advancement or reputation; or (3) identity of ‘subjects’ can be readily ascertained and an IRB conducts a limited review.

Research designed to evaluate or improve public benefit or service programmes (e.g. procedures for obtaining benefits under these programmes, alternatives to those programmes, changes in levels of payments for benefits or services, etc.) is also considered exempt.

Although research may be categorised as ‘exempt’, it may nevertheless be subject to IRB approval or review if, for example, it involves prisoners, pregnant women, children or members of other protected classes or vulnerable groups. In addition to the regulations set forth at 45 CFR 46 with oversight by the Office for Human Research Protections, research in the United States may also be regulated by the Food and Drug Administration, National Institutes of Health, and other sponsors or state or local governments. Jurisdictional issues and sources of funding may also play a role in the determinations of exempt status.

### Netherlands

In the Netherlands, medical/scientific research that exposes participants to procedures/treatments, or requires participants to follow rules of behaviour, falls under the scope of the Medical Research Involving Human Subjects Act (WMO) and must undergo review by an accredited Medical Research Ethics Committee or the CCMO.

Studies involving human participants are not subject to the WMO if persons are not exposed to procedures/treatment or are not required to follow a certain behavioural strategy. For example, research is exempt if a participant is not physically involved in the research and only their previously collected data is being used. Retrospective research, such as file research or research with residual tissue, is therefore considered exempt [26]. However, the Dutch government is currently preparing legislation that introduces an obligatory review of secondary use of tissue for research to be carried out by the same accredited Medical Research Ethics Committees.

In some cases, it may be unclear what research activities fall within or outside of the scope of the WMO. Therefore, the CCMO has set up guidelines considering the scope of the law. The guidelines make clear that a study in which a participant provides a urine sample only once is not subject to the WMO (i.e. that study is considered exempt). However, if the participant were required to repeatedly provide urine samples over several weeks, that research would fall within the scope of the law (i.e. require ethics review) as the participant would have to follow a specific behavioural strategy [26].

On the other hand, the CCMO offers an example of a randomised controlled trial that would be exempt from the requirement for ethics review, that of a study randomising patients to the use of mattress A or mattress B in a hospital. This would be exempt from ethics review as it is not considered a change of normal hospital procedures [26].

Research consisting of interviews or questionnaires falls outside of the scope of the WMO and is exempt from ethics review unless it imposes a rule of behaviour (e.g. it needs to be conducted daily over several days). However, if the topic is sensitive (e.g. it pertains to suicide, mental illness, etc.) this research is no longer exempt [31].

Post-marketing studies, such as those collecting data on side effects of medication that is already available on the market, are also considered exempt [31]. However, this type of research is not completely free of assessing its medical-ethical (and legal) aspects – guidelines (‘self-regulation’) provide for a proper (i.e. lighter) review system.

## Discussion

All four countries exempt from ethics review at least some types of human health and medical research. The broadest set of exemptions exist in the Netherlands and the narrowest in Australia. All countries exempt research on existing data or specimens, provided they are non-identifiable; three countries exempt questionnaire or survey research, albeit subject to stipulated conditions, for example, minimal inconvenience.

Historically, ethics review has been important to ensure an appropriate balance of potential benefits and risks and informed consent for higher-risk research. Although one of the most common exemptions is research involving non-identifiable existing data or specimens, the issue of non-identifiability requires careful management, as considerable risks may arise from re-identification, e.g. in context of genetic studies [32]. Nevertheless, much low-risk research – for example, surveys, quality improvement projects, clinical audits – has been caught in the ethics assessment net, with different nets in different countries, perhaps partly driven by the lack of clear boundaries between them [6, 33].

This initial comparison of international variations in research ethics reviews documents these variations but explaining them confronts the challenges associated with any international comparison – that is, difficulties in comparing research regulation within differing healthcare systems and settings. These reflect varying sociocultural values and differences in the histories that have driven evolution of research ethics regulation and legislation in each country. Moreover, although each of the documents analysed here is a key national-level document pertaining to ethical issues around human health and medical research, they are not identical in scope. Inevitably, therefore, the findings presented here are simplified and omit some of the complexities of each system. Including more countries in the comparison would almost certainly have yielded evidence of yet more diversity than that we have documented. The selection of these particular countries was driven by pragmatic considerations, including availability of expertise and English-language documentation; however, it is noteworthy that even this limited sample establishes considerable variety in international practices.

The evidence of waste resulting from hyper-regulation of clinical and health research that motivated this research demands greater accountability to the public among research regulators. Their work requires auditing just as this is an expectation of other professionals.

The variations in exemptions from ‘full’ ethics review we have identified suggest opportunities to extend documentation of international and intranational differences and proposals for streamlining of ethics review processes. Although innovation in the area of ethics review may pose considerable challenge, some innovations in this area have recently been implemented and described [34–36]; for example, in a review of its research ethics processes, Médecins Sans Frontières developed four innovative practices, namely (1) introduction of a policy exempting a posteriori analysis of routinely collected data; (2) the preapproval of ‘emergency’ protocols; (3) general ethics approval of ‘routine’ surveys; and (4) evaluating the impact of approved studies [36]. Nevertheless, the aforementioned criticisms of current practices in ethics reviews – that they impede research, fail to protect participants, are burdensome and inconsistent across sites – coupled with the calls to investigate and implement ‘alternative’ models of review, make clear that further innovation and evaluation is still much needed [36, 37].

It would be helpful if the bodies in charge of providing guidance on research ethics could provide clear and transparent rationale underpinning each exemption. The Netherlands provides an excellent example of this, explicitly stipulating that exposing participants to treatment, or requiring them to follow behavioural rules, are the determinants of whether research requires ethics review. Clarity and transparency in the rationales underpinning exemptions could assist research ethics committee decision-making, potentially freeing up the time and resources of both ethics committee members (to focus on higher risk projects) and researchers (to conduct their research).

Exemptions identified here could also be considered by countries that do not presently exempt these types of research. For example, the United Kingdom’s exemption of research involving NHS staff acting in their professional roles – or more broadly, research involving healthcare providers and experts querying their expertise – could be considered for adoption in other countries. For example, our identification of the paucity of exemptions in Australia as compared to the other countries has so far spurred two initiatives – a petition to the Australian Government to seek a national inquiry to streamline and improve research ethics and governance in Australia [38], and a national survey (recently completed) of researchers and ethics committee members. The survey helped to identify the extent to which current research ethics rules lead researchers to modify or forego research projects as well as the reasons for potentially exempting, in Australia, some of the exemptions identified here. The survey results will be provided to the NHMRC for its next planned revision of the National Statement on Ethical Conduct in Human Research.

Finally, where a full exemption is considered inappropriate, there is a wide range of options that could decrease some of the burden of ethics reviews, including partial exemption or expedited review. These options are often identified in ethics guidance documents (e.g. NHMRC) but they remain underused, as ethics review practices are more demanding than that required by regulations [5]. One example of this ‘partial’ approach is the Proportionate Review process used by the United Kingdom’s Health Research Authority [39], which has reduced review times for relevant projects by a third [40]. Eligible for Proportionate Review are those studies that pose minimal risk, burden or intrusion to participants, including research with anonymously collected data or tissue, questionnaires or interviews that do not include highly sensitive areas, or research surveying the safety or efficacy of established non-drug treatments, and excluding research involving prisoners, adults lacking capacity to consent or clinical trials of investigational medicinal products [39]. Studies that are judged appropriate for Proportionate Review using the No Material Ethical Issues Tool can be reviewed remotely by three REC members (with at least 6 months service on a research ethics committee) with the aim of providing a decision to the researchers within 21 calendar days, although the Health Research Authority has recently piloted reducing this time to 13 working days [40]. RECs completing a proportionate review are able to promote a proportionate review project to full review if they think there are significant ethical issues. Consultations are ongoing as to whether this review process could be expanded to a wider range of projects.

## Conclusion

Given the considerable costs and burdens on researchers and ethics committees, it would seem worthwhile to develop and provide clearer guidance on exemptions from full ethics review, illustrated with examples derived from multiple jurisdictions, with transparent underpinning rationales. We hope the examples provided here may be a motivator to free the negligible and low risk research from the “*shadow of protracted ethics review*” [33].

## Data Availability

Not applicable.

## Abbreviations

45 CFR 46: Code of Federal Regulations Title 45: Public Welfare, part 46
CCMO: Central Committee on Research Involving Humans
IRB: Institutional Review Board
NHMRC: National Health and Medical Research Council
NHS: National Health Service
REC: Research Ethics Committee
WMO: Medical Research Involving Human Subjects Act
